# Tuning the structure of the Josephson vortex lattice in Bi_2_Sr_2_CaCu_2_O_8+δ_ single crystals with pancake vortices

**DOI:** 10.1038/s41598-018-28681-7

**Published:** 2018-07-19

**Authors:** P. J. Curran, H. A. Mohammed, S. J. Bending, A. E. Koshelev, Y. Tsuchiya, T. Tamegai

**Affiliations:** 1 Department of Physics, University of Bath, Claverton Down, Bath, BA2 7AY UK; 2 Department of Physics, University of Kirkuk, Kirkuk, Iraq; 3 Materials Science Division, Argonne National Laboratory, Argonne, Illinois 60439 USA; 4 Department of Applied Physics, The University of Tokyo, Hongo, Bunkyo-ku, Tokyo 113-8656 Japan; 5 Department of Electrical Engineering, Nagoya University, Nagoya, Aichi 4648603 Japan

## Abstract

In extremely anisotropic cuprate superconductors a lattice of stacks of pancake vortices nucleates when a magnetic field is applied perpendicular to the copper oxide layers, while an orthogonal lattice of highly elliptical Josephson vortices forms when the applied field is parallel to the layers. Under tilted magnetic fields these sublattices can interact in complex ways to form systems of vortex chains and composite vortex lattices. Here we have used high-resolution scanning Hall microscopy (SHM) to map the rich tilted-field vortex phase diagram in an underdoped Bi_2_Sr_2_CaCu_2_O_8+δ_ single crystal. We find that the Josephson vortex lattice spacing has an unexpected non-monotonic dependence on the pancake vortex density reflecting the delicate balance between attractive and repulsive vortex interactions, and actually undergoes a field-driven structural transformation with increasing out-of-plane fields. We also identify particularly stable composite structures composed of vortex chains separated by an integer number of rows of interstitial pancake vortex stacks and are able to establish the precise evolution of vortex-chain phases as the out-of-plane field is increased at small in-plane fields. Our results are in good semi-quantitative agreement with theoretical models and could enable the development of vortex ratchets and lenses based on the interactions between Josephson and pancake vortices.

## Introduction

Superconductivity in the ‘high-temperature’ cuprate Bi_2_Sr_2_CaCu_2_O_8+δ_ (BSCCO-2212) is hosted in CuO_2_ bilayers lying parallel to the a-b crystallographic planes that are only weakly coupled along the perpendicular c-axis. The strong resulting crystalline anisotropy leads to very large magnetic anisotropy parameters, γ = λ_c_/λ_ab_ (where λ_c_ and λ_ab_ are the c-axis and a-b plane magnetic field penetration depth, respectively), that can reach values of several hundred depending on the oxygen doping level of the crystal. A magnetic field applied perpendicular to the CuO_2_ bilayers leads to the nucleation of stacks of two-dimensional ‘pancake vortices’ (PVs)^1,2^ whose supercurrents flow within the CuO_2_ planes and that interact to form a well-ordered triangular Abrikosov lattice in high-quality single crystals. If, on the other hand, the field is applied in the a-b plane, Josephson vortices (JVs)^1,2^ nucleate centred on the spaces between CuO_2_ bilayers with circulating currents composed of high current density parts within the copper oxide planes and weak Josephson-like components between them. This anisotropic current distribution leads to very anisotropic JV-JV interactions and a highly stretched rhombic JV lattice.

**Figure 1:**
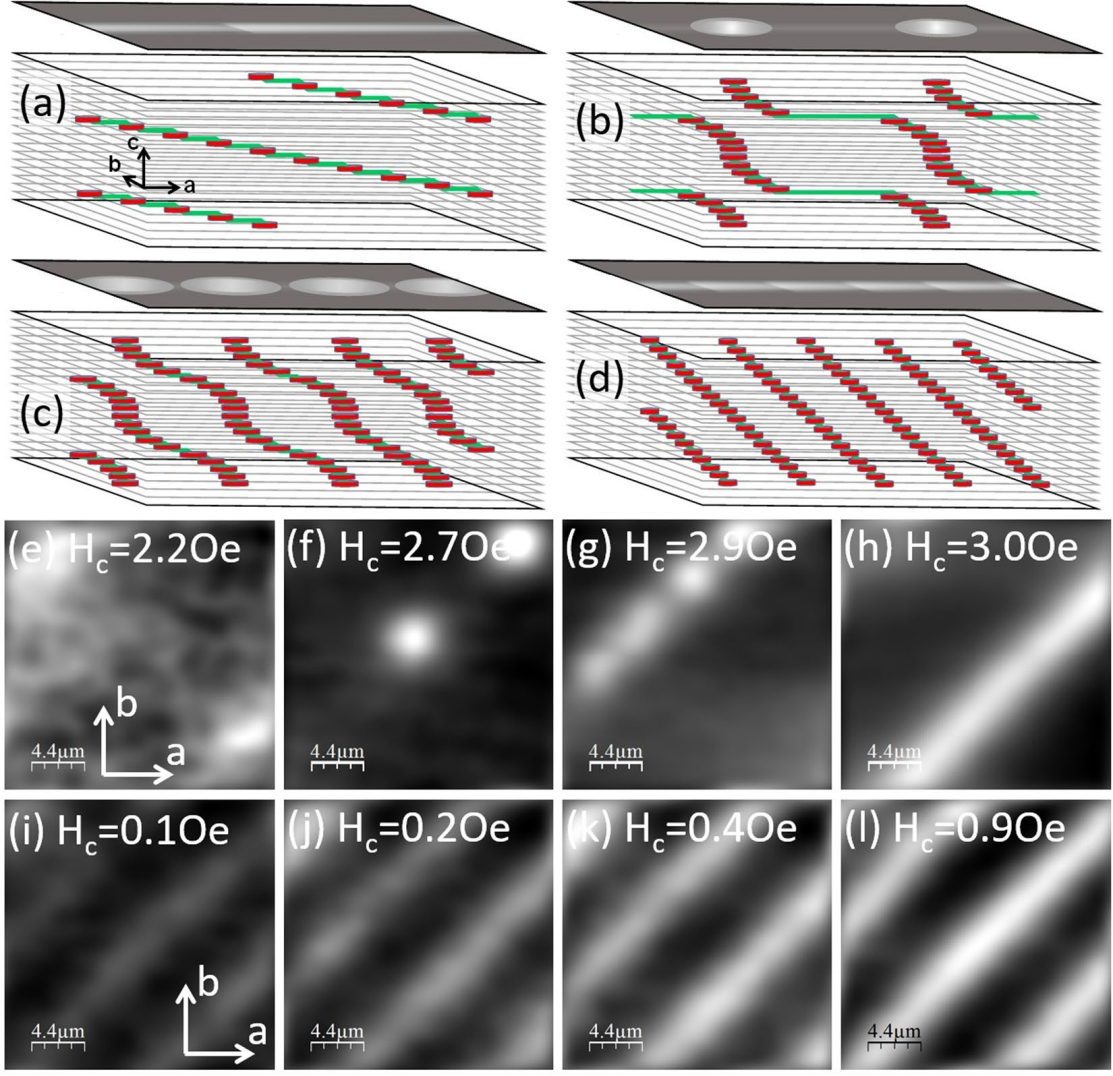
Structure of vortex-chain phases at low out-of-plane magnetic fields. (**a**–**d**) 3D sketches of predicted vortex-chain states as the out-of-plane field is increased at fixed in-plane field. Red disks represent pancake vortices and horizontal green lines represent Josephson vortices. The in-plane field lies along the chain direction, labelled as the a-axis in this case. The map above each image shows the expected B_c_ distribution as measured by our scanning Hall probe at the crystal surface. (**a**) Kinked tilted vortices, (**b**) crossing pancake-stacks, (**c**) modulated tilted vortices and (**d**) straight tilted vortices. (**e**–**h**) Top-down view SHM images of vortex chains at T = 85 K and H_ab_ = 30 Oe showing their evolution as the out-of-plane field is increased from 2.2 Oe to 3.0 Oe (grayscale spans (**e**) 0.13 G, (**f**) 0.97 G, (**g**) 0.94 G, (**h**) 1.92 G). (**i**–**l**) Top-down view SHM images of vortex chains at T = 85 K and H_ab_ = 200 Oe showing their evolution as the out-of-plane field is increased from 0.1 Oe to 0.9 Oe (grayscale spans (**i**) 0.50 G, (**j**) 0.78 G, (**k**) 0.93 G, (**l**) 1.2 G). Field of view ≈22 × 22 μm^2^.

If the field is instead applied at an arbitrary ‘tilt’ angle away from the crystallographic c-axis, the very high anisotropy in BSCCO-2212 leads to a wide variety of possible groundstates including kinked lattices^3–6^, tilted vortex chains^7–9^ and coexisting orthogonal PV and JV lattices^4,10^. For low out-of-plane fields (B_c_ ≈ 0–2 G) all pancake vortices interact strongly with stacks of Josephson vortices over a wide range of in-plane fields (B_ab_ ≈ 10–650 G) in so-called vortex-chains phases. This limit has been theoretically investigated both analytically and numerically^11^, and the rich lattice structures that can arise due to the combined magnetic and Josephson interactions between pancake vortices in different layers were elucidated. The key parameter that quantifies the relative strength of these two interactions is ${\rm{\alpha }}={{\rm{\lambda }}}_{{\rm{ab}}}/{{\rm{\lambda }}}_{{\rm{J}}}$, where ${{\rm{\lambda }}}_{{\rm{J}}}={\rm{\gamma }}s$ is the Josephson length (s = 1.5 nm is the c-axis spacing between CuO_2_ bilayers). For values of α in the range 0.5 ≤ α ≤ 0.65 a very small c-axis magnetic field is predicted to penetrate in the form of *kinked tilted vortices*, composed of single pancake vortex ‘kinks’ separated by quite long segments of Josephson vortex^11^. Upon increasing H_c_ the system undergoes a first-order transition to a phase of *pancake-stack chains*, whereby mostly vertical stacks of pancake vortices cross a stack of Josephson vortices, and PVs close to JV cores are displaced along the chain by the JV currents. PV kinks repel one another while crossing PV-stacks attract at long distances, and this leads to a large associated jump in pancake density at the transition to a state with a maximum equilibrium spacing near to the separation where the PV-stack interaction has a minimum. At higher out-of-plane fields crossing PV-stacks smoothly evolve into *modulated tilted vortices*, when most pancake vortices are quite strongly displaced from a linear average distribution. Finally, at higher H_c_ the system transforms via a second-order phase transition to *straight tilted vortices*. These four distinct vortex-chain phases are sketched in panels (a)–(d) of Fig. 1 with maps of the expected B_c_ distribution as measured by our Hall probe at the crystal surface shown above each. The exact sequence of phases depends on the specific value of B_ab_, and above a critical in-plane field the crossing pancake-chain state no longer arises and a first-order transition from kinked tilted vortices to tilted straight vortices is predicted. If the out-of-plane field is increased still further, free interstitial pancake vortex stacks nucleate between the vortex-chains and *composite vortex matter* phases are formed. These have many competing attractive and repulsive interactions and no theoretical work yet exists which attempts to fully understand the complex resulting vortex structures that form, in particular, the role of thermal fluctuations is not fully understood. Previous vortex imaging work^12–17^ has only experimentally investigated a tiny part of the available phase space, and here we describe an extensive vortex imaging survey in an underdoped BSCCO-2212 single crystal which allows us to develop a detailed vortex-matter phase diagram as a function of in-plane and out-of-plane fields. Our work provides important insights into the properties of vortex matter in highly anisotropic cuprate superconductors under tilted magnetic fields and will inform the development of first generation high-temperature superconductor wires based on them.

## Results

The images presented here have been captured at many different places above the surface of the underdoped BSCCO-2212 single crystal with T_c_ = 89 K and our observations do not depend significantly on the location. Our 2D Hall probe is only sensitive to the component of magnetic induction perpendicular to the a-b crystal surface and samples fields from pancake vortices to a depth of about λ_ab_ with a weighting that decays exponentially with depth. Before each measurement the desired out-of-plane field is applied at T = 85 K and an in-plane field ‘dithering’ protocol is employed to equilibrate the vortex distribution (see methods). Note that tilted vortices are expected to have a weak asymmetric field modulation along their length but this lies well below the measurement resolution of our Hall sensor (B_min_ ~ 0.1 G with the 100 Hz measurement bandwidth used) and they appear uniform in our images (see Supplementary Materials).

### Vortex-chain phases

We focus first on the low out-of-plane field regime when all pancake vortices interact strongly with Josephson vortices in so-called vortex-chain phases. At these fields all PVs intersect stacks of JVs and there are no free interstitial PVs between the chains. For our underdoped BSCCO-2212 crystal we estimate that ${\rm{\alpha }}={{\rm{\lambda }}}_{{\rm{ab}}}/({\rm{\gamma }}s)\cong 0.53\pm 0.02$, placing our samples in the 0.5 ≤ α ≤ 0.65 regime discussed in ref.^11^. Here we have used the ‘intrinsic’ anisotropy, γ = 840 ± 20, measured from the chain spacing at B_c_ ~ 0 (see Supplementary Information) and λ_ab_ = 660 ± 20 nm as estimated from underdoped samples in ref.^18^ with the same T_c_, assuming a two-fluid temperature dependence ${{\rm{\lambda }}}_{{\rm{ab}}}({\rm{T}})={{\rm{\lambda }}}_{{\rm{ab}}}(0)/\sqrt{1-{({\rm{T}}/{{\rm{T}}}_{{\rm{c}}})}^{4}}$. For moderate in-plane fields above ~10 Oe, it is predicted that vortex-chain states will evolve from kinked tilted vortices to straight tilted vortices with increasing c-axis magnetic field, as discussed in the introduction. Figure 1(e–h) show SHM images of PVs in our crystal at a fixed in-plane field of H_ab_ = 30 Oe and four out-of-plane fields in the range 2.2–3.0 Oe. When PVs have just penetrated the sample at H_c_ = 2.2 Oe, we resolve two very faint stripes with amplitude ≈0.1 G running diagonally across the image, and we associate these stripes with the predicted kinked tilted vortex phase of Fig. 1(a) composed of single PV kinks separated by ≈1 μm long segments of JV at this value of B_c_. Upon increasing the out-of-plane field to 2.7 Oe, two bright well-resolved crossing PV-stacks form with approximately ten times larger amplitude (~0.94 G), consistent with the predicted first-order transition. At a higher c-axis field of 2.9 Oe, we observe a rather inhomogenous state along the chain composed of two well-resolved bright PV-stacks at the top and two much broader structures below. We tentatively identify the latter with the onset of the modulated tilted vortices predicted in this regime. For H_c_ ≥ 3 Oe, we observe only bright structureless stripes which we associate with the expected straight tilted vortex phase. In contrast to this behaviour at low in-plane fields, Fig. 1(i–l) show images captured at a larger fixed in-plane field of H_ab_ = 200 Oe when we only observe unstructured stripes for all out-of-plane fields in the range 0.1 Oe ≤ H_c_ ≤ 5 Oe. This is also consistent with theoretical work^11^ which predicts that above a critical in-plane field (estimated to be H_ab_ ≈ 65 Oe for our sample) only tilted chains are realized and the system jumps from kinked tilted vortices at low fields to straight tilted vortices at high fields. However, the calculations also predict a jump in the PV density at this transition, while empirically the chain amplitude in our images appears to increase smoothly from 0.16 G at first penetration to a maximum of 1.6 G at H_c_ = 3 Oe.

### Composite vortex phases at high out-of-plane fields

**Figure 2:**
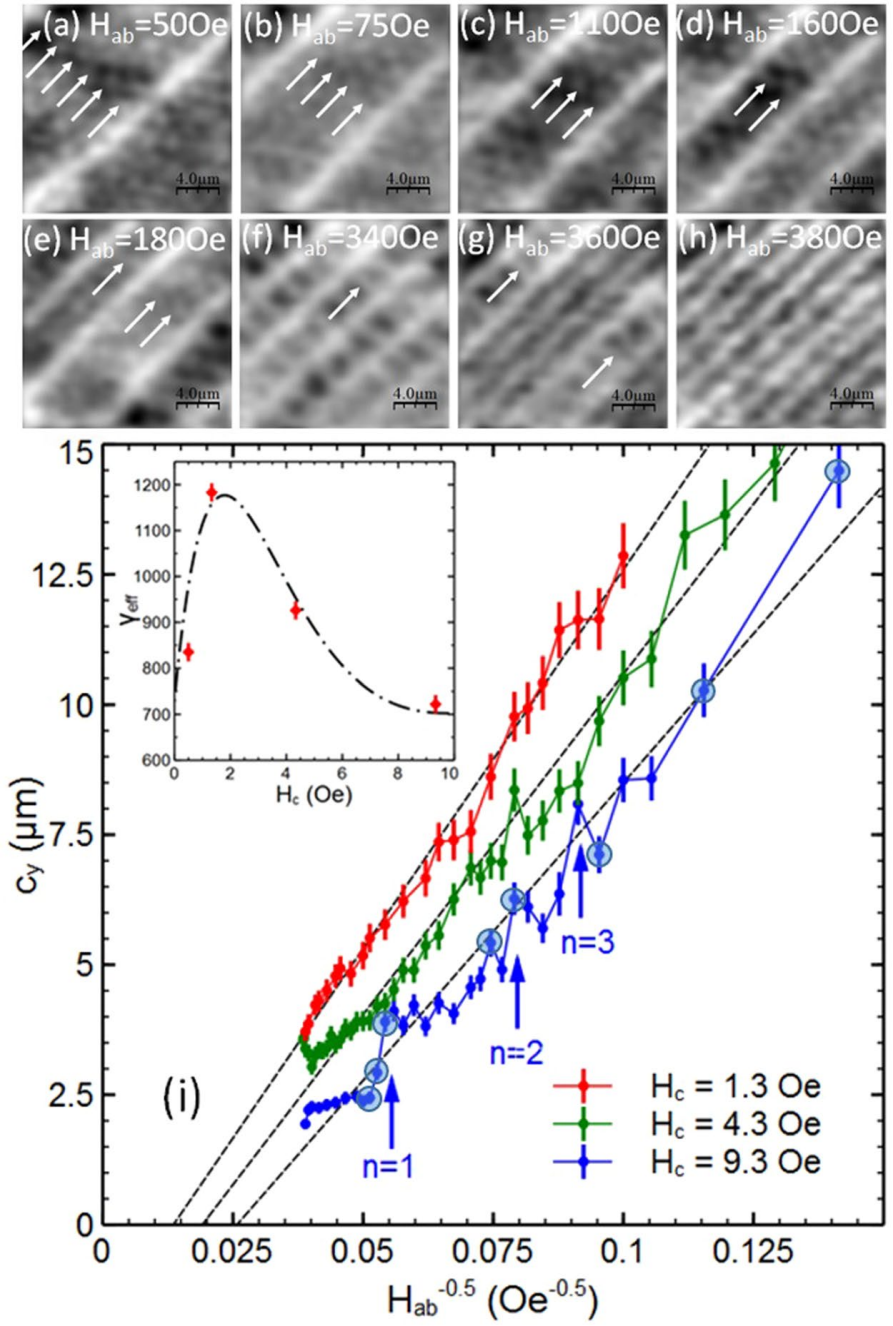
Behaviour of vortex-chain states as a function of in-plane magnetic field. (**a**–**h**) SHM images of vortex chains (bright lines) and interstitial pancake vortex stacks (feint dots) at T = 85 K and H_c_ = 9.3 Oe, and the indicated values of H_ab_. The vortex chain spacing decreases as the in-plane field is increased and the number of rows of interstitial pancake vortex stacks (indicated by arrows) decreases. Field of view ≈22 × 22 μm^2^. (**i**) The mean vortex-chain spacing, c_y_, as a function of $1/\sqrt{{{\rm{H}}}_{{\rm{ab}}}}$ at T = 85 K and three different values of out-of-plane field. Dashed lines are linear regression fits to the data used to extract the effective anisotropy, γ_eff_. Vertical arrows indicate peaks in the H_c_ = 9.3 Oe curve corresponding to particularly stable composite vortex states with n rows of interstitial pancake vortex stacks between vortex-chains. Data points surrounded by larger circles relate to the specific SHM images shown in (**a**–**h**). The inset shows the measured out-of-plane magnetic field dependence of γ_eff_. The dot-dashed line is a guide to the eye.

We now turn our attention to measurements at high out-of-plane magnetic fields when composite vortex phases exist, and interstitial pancake vortex stacks nucleate *between* the vortex-chains. Figure 2(a–h) show SHM images captured at a fixed out-of-plane field, H_c_ = 9.3 Oe, as the in-plane field is increased from 50 Oe to 380 Oe. Vortex chains can easily be recognised as they are brighter, having higher pancake densities due to the attractive mutual PV/JV interaction. All the frames except Fig. 2(h) also contain fainter interstitial pancake vortex stacks between the chains which are clearly organised into rows (indicated by white arrows) running parallel to the adjacent diagonal vortex chains. Since the vortex-chain spacing decreases with increasing in-plane field, the number of PV-stack rows is seen to decrease at higher fields until they become squeezed out entirely at H_ab_ = 380 Oe when all pancake vortices are incorporated into vortex-chains. Figure 2(d) and (f) show two especially well-ordered structures which contain two and one PV-stack rows, respectively, and we will demonstrate later that these represent particularly stable composite vortex configurations.

Figure 2(i) shows results of the analysis of the average vortex-chain spacing in images like Fig. 2(a–h) at three different fixed values of the out-of-plane field. The chain spacing, c_y_, has been plotted against $1/\sqrt{{{\rm{H}}}_{{\rm{ab}}}}$ to reflect the expected magnetic field dependence of the unperturbed (H_c_ = 0) Josephson lattice, ${{\rm{c}}}_{{\rm{y}}}=\sqrt{{{\rm{\gamma }}}_{{\rm{eff}}}{{\rm{\Phi }}}_{0}/{{\rm{\beta }}{\rm{B}}}_{{\rm{ab}}}}$, where ${\rm{\beta }}=2/\sqrt{3}$ or $2\sqrt{3}$ is a coefficient that selects one of two normally degenerate Josephson vortex structures and γ_eff_ is the effective lattice anisotropy. Note that deviations are expected from the behaviour ${{\rm{c}}}_{{\rm{y}}}\,{\rm{\alpha }}\,1/\sqrt{{{\rm{B}}}_{{\rm{ab}}}}$ at very high in-plane fields because the layered nature of BSCCO-2212 crystals breaks the continuous London approximation; in this regime c_y_(B_ab_) should initially peel away from the fit lines in Fig. 2(i) towards the horizontal axis^19^. Assuming ${\rm{\beta }}=2/\sqrt{3}$ (known to be the ground state at low H_c_^10^), the reduction in slope at high out-of-plane fields can be interpreted as a strong decrease in the effective anisotropy (γ_eff_ = 1180 at 1.3 Oe, 930 at 4.3 Oe, 720 at 9.3 Oe). Furthermore, if we estimate the anisotropy from the chain spacing when stripes can first be resolved in the kinked tilted lattice phase at very low out-of-plane fields (B_c_ ≃ 0), we find a value of γ_eff_ = 840. The dependence of the effective anisotropy on H_c_ is summarised in the inset of Fig. 2(i), where we see that it exhibits an unexpected non-monotonic dependence, increasing sharply up to H_c_ ~ 1–2 Oe due to the attractive JV-PV interaction followed by a more gradual decrease driven by PV-PV repulsion (see discussion section). One other striking feature of Fig. 2(i) is the presence of growing deviations from the expected linear behaviour exhibited at high H_c_, in particular the pronounced peaks seen at H_c_ = 9.3 Oe. These have been labelled with indices, n, corresponding to the number of rows of interstitial PV-stacks present in these highly ordered composite vortex configurations. As indicated above, these represent particularly stable states when the chains are pushed further apart by the PV-stack rows in between.

**Figure 3:**
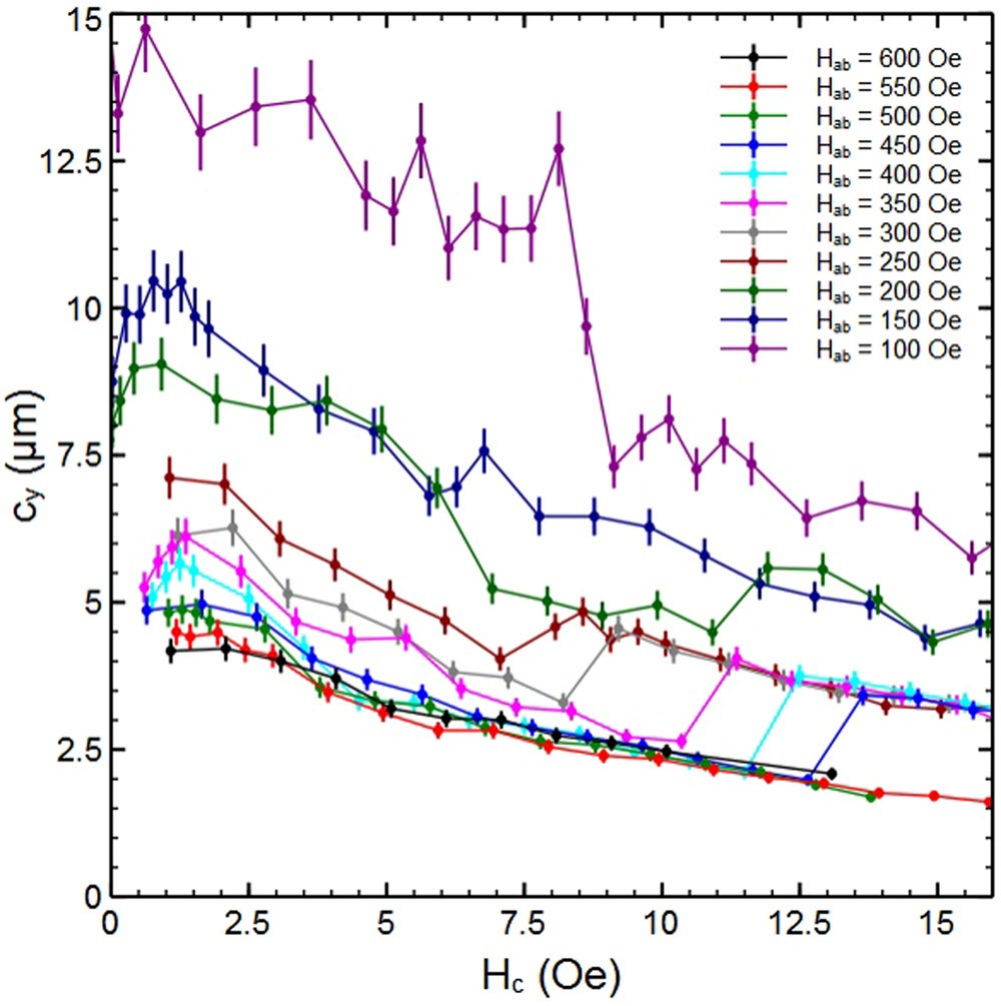
Behaviour of vortex-chain states as a function of out-of-plane magnetic field. The mean vortex-chain spacing, c_y_, as a function of H_c_ at several different values of fixed in-plane field, H_ab_, and T = 85 K. The curves show a systematic reduction in c_y_ with increasing H_c_ due to a field-driven transformation of the Josephson vortex lattice. Superimposed on this are abrupt jumps to larger vortex chain spacing that correspond to the formation of particularly stable composite vortex states with integer numbers of rows of interstitial PV stacks.

Since c_y_ is not expected to depend strongly on H_c_, a more insightful way to present these data is to plot the vortex-chain spacing versus out-of-plane magnetic field at fixed H_ab_ as shown in Fig. 3. While the expected trend for the chain spacing to reduce with increasing in-plane field is broadly observed there are several quite abrupt jumps in the data that obscure it, and there appears to be a tendency for c_y_ to saturate to a value that is almost independent of in-plane field for H_ab_ > 400 Oe. We can also now resolve that the chain spacing initially increases at very small out-of-plane fields up to about 1–1.5 Oe before falling again as the field is increased further.

### Field-driven structural transformation of the Josephson vortex lattice

**Figure 4:**
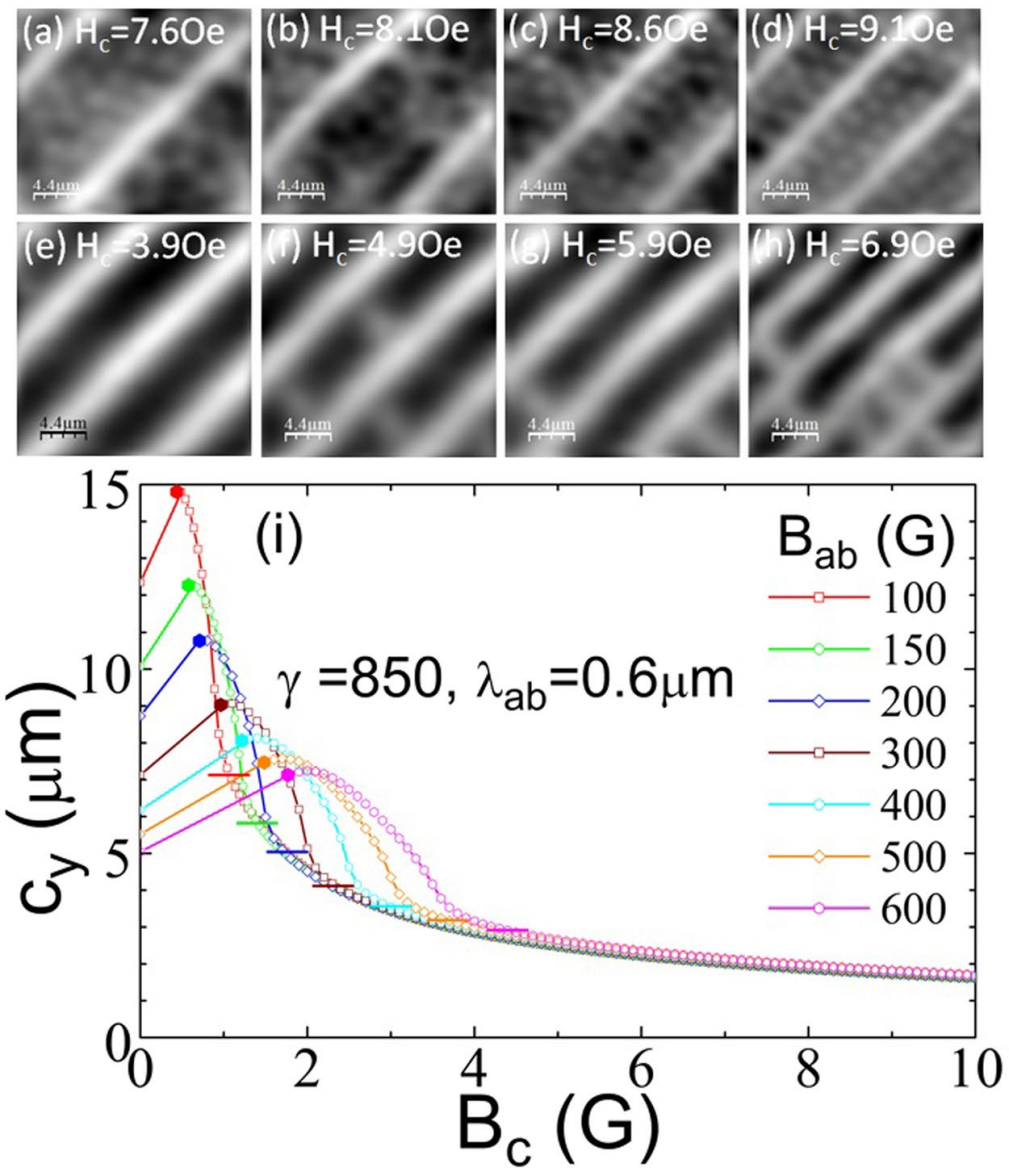
Field-driven transformation of the Josephson vortex lattice. (**a**–**d**) SHM images at T = 85 K and H_ab_ = 100 Oe, and the indicated values of H_c_ showing a very abrupt reduction in vortex chain spacing over the range H_c_ = 8.1–9.1 Oe. (**e**–**h**) SHM images at T = 85 K and H_ab_ = 200 Oe, and the indicated values of H_c_ showing a much more gradual reduction in vortex chain spacing over the range H_c_ = 3.9–6.9 Oe. Field of view ≈22×19 μm^2^ (**a**–**d**) and ≈22×22 μm^2^ (**e**–**h**). (**i**) The theoretically computed dependencies c_y_(B_c_) at several values of in-plane magnetic induction, B_ab_, showing a reduction in the vortex chain spacing for increasing B_c_ due to a field-driven transformation of the Josephson vortex lattice (see text). Initial penetration of the PV stacks triggers a jumplike increase of c_y_ due to the attractive PV-JV crossing energy. Further rapid decrease is caused by a structural transformation of the JV lattice driven by PV-PV repulsion. This decrease stops when c_y_ reaches the second stable lattice parameter c_y2_ marked by the horizontal bars.

Figure 3 clearly shows that the chain spacing at fixed H_ab_ generally decreases quite smoothly as we increase the out-of-plane field above ~1.5 Oe. The main exception to this behaviour occurs in the data at H_ab_ = 100 Oe, where we see a rather abrupt ≈40% drop in c_y_ in the narrow range H_c_ = 8–9 Oe, as illustrated by the sequence of SHM images shown in Fig. 4(a–d). Figure 4(e–h) at H_ab_ = 200 Oe show the more common higher field behaviour when the chain spacing more gradually reduces by a similar fraction in the broader range H_c_ = 4–7 Oe. Figure 4(i) shows the results of calculations of the chain spacing as a function of B_c_ for several values of B_ab_ where the on-chain mutual PV repulsion, which competes with the PV-JV attraction, has been taken into account. This model qualitatively captures the experimental behaviour of Fig. 3 including the increase in chain spacing at very low out-of-plane fields. It also shows a more rapid reduction in chain spacing with H_c_ at lower values of in-plane field, and c_y_ is seen to saturate to a value that is approximately independent of B_ab_ at large out-of-plane field. A careful analysis of these numerical results reveals that the increasing PV density actually drives a structural transformation of the underlying JV lattice. Within London theory the non-interacting JV lattice in BSCCO-2212 exhibits two energetically degenerate structures with JV stack spacings ${{\rm{c}}}_{{\rm{y}}1}=\sqrt{\sqrt{3}{\gamma }_{{\rm{eff}}}{{\rm{\Phi }}}_{0}/(2\,{{\rm{B}}}_{{\rm{ab}}})}$ and ${{\rm{c}}}_{{\rm{y}}2}=\sqrt{{\gamma }_{{\rm{eff}}}{{\rm{\Phi }}}_{0}/(2\sqrt{3}\,{{\rm{B}}}_{{\rm{ab}}})}$, respectively. For small out-of-plane fields it is known that the state with the largest JV stack spacing, c_y1_, has the lowest energy because it leads to the largest number of JV-PV crossing points^10^. However, we find that at higher PV densities a transformation to the state with the smaller stack spacing, c_y2_, takes place since this reduces the on-chain PV-PV spacing and hence the-PV-PV repulsion.

### Special stable composite vortex lattice states

**Figure 5:**
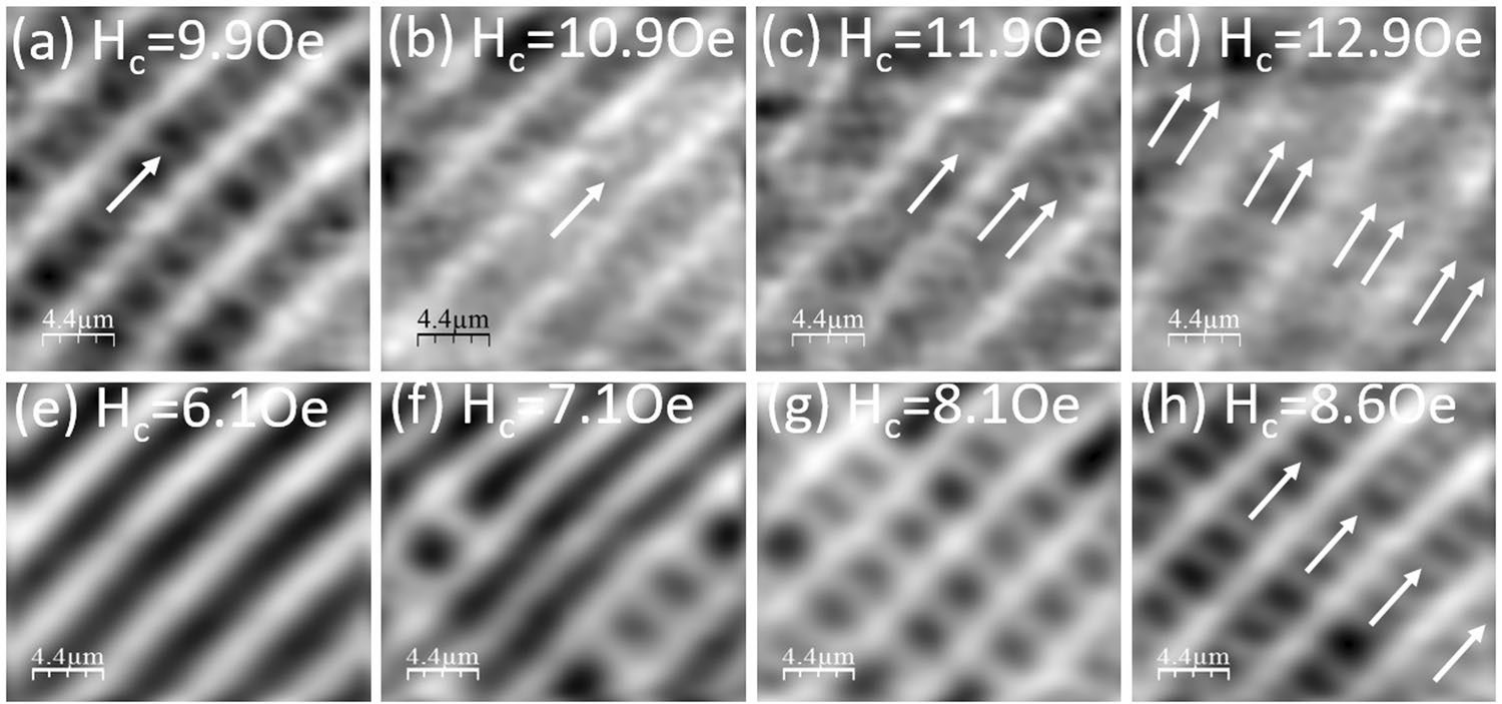
Stability of composite vortex lattice states. (**a**–**d**) SHM images at T = 85 K and H_ab_ = 200 Oe, and the indicated values of H_c_ illustrating the formation of a very stable composite vortex state with exactly two rows of interstitial pancake vortices (indicated by arrows). (**e**–**h**) SHM images at T = 85 K and H_ab_ = 250 Oe, and the indicated values of H_c_ illustrating the formation of a very stable composite state with exactly one row of interstitial PVs. Field of view ≈ 22 × 22 μm^2^ (**a**–**d**) and ≈22 × 19 μm^2^ (**e**–**h**).

In addition to a reduction in chain spacing at high out-of-plane fields, Fig. 3 also shows abrupt step-like increases in c_y_ at H_c_ ≈ 12 Oe and H_ab_ = 200 Oe, and at various out-of-plane fields in the range 6–13 Oe for in-plane fields spanning 150–450 Oe. Figure 5(a–d) show SHM images at H_ab_ = 200 Oe which reveal that the abrupt increase in chain spacing at H_c_ ~ 12 Oe is associated with the formation of a stable composite vortex state with exactly two rows (n = 2) of free PV-stacks between the vortex-chains. Figure 5(e–h) at H_ab_ = 250 Oe show that the jump in chain spacing at H_c_ ≈ 8 Oe corresponds to the formation of a stable composite state with exactly one row (n = 1) of free PVs between vortex chains. The same is true for several n = 1 related jumps occuring in the range H_ab_ = 150–450 Oe. Surprisingly, we are unable to track this jump at H_ab_ > 450 Oe, even though the signal-to-noise in our SHM images at these very high fields should still be high enough to resolve it.

### Proposed composite vortex matter phase diagram

**Figure 6:**
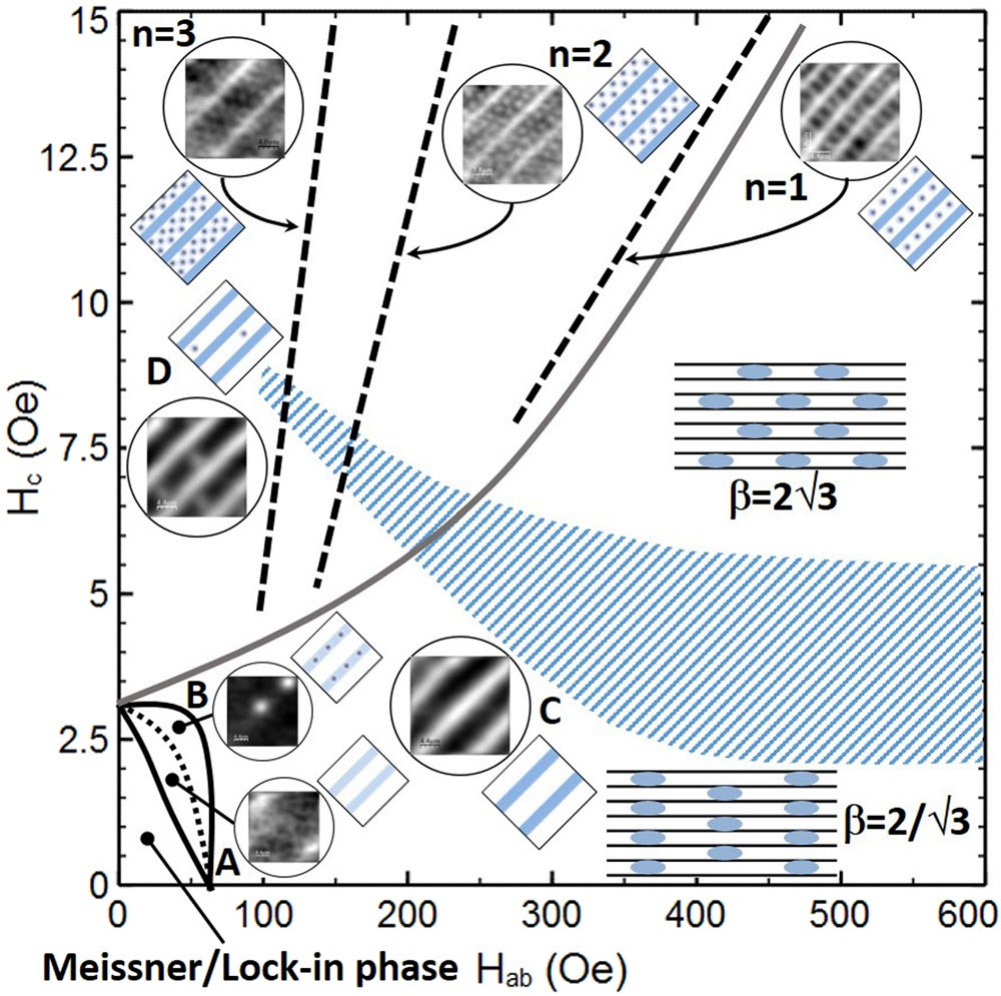
Proposed phase diagram of composite lattice vortex matter. Proposed vortex-matter phase diagram as a function of out-of-plane and in-plane fields based on our experimental findings at T = 85 K. The heavy grey line divides the diagram into regions with free interstitial pancake vortices (upper left) and those without (lower right). The region of diagonal hatching indicates the range of values of H_c_ over which the field-driven Josephson vortex lattice transition takes place. Square graphics show sketches of the projected pancake vortex structures in the indicated regions. Square graphics surrounded by circles show typical experimental SHM images of pancake vortices that correspond to these sketches. Rectangular graphics show side views of the two Josephson vortex lattice structures with different values of β (see text).

Figure 6 shows the proposed phase diagram based on our experimental findings for composite vortex matter in our underdoped BSCCO-2212 single crystal at T = 85 K as a function of in-plane and out-of-plane magnetic fields, supplemented with representative experimental images and corresponding sketches of the vortex structures forming in different regimes. The grey line running approximately diagonally across the diagram separates regions of pure vortex-chains (bottom right) from those containing free interstitial PV-stacks between the chains (top left). The area of diagonal hatching represents the crossover region between the two distinct Josephson vortex lattice structures with ${\rm{\beta }}=2/\sqrt{3}$ (small H_c_) and ${\rm{\beta }}=2\sqrt{3}$ (large H_c_). As the out-of-plane field is increased at H_ab_ < 65 Oe, we first encounter a Meissner region with no pancake vortices, followed by the penetration of PVs in kinked tilted vortices (region A). As H_c_ is increased still further, there is a first-order transition to a system of ‘crossing’ pancake stacks (region B) which distort into modulated tilted vortices and then transform into straight tilted vortices (region C). At still higher out-of-plane fields free interstitial pancake vortex stacks nucleate between vortex-chains (region D). The location of particularly stable composite vortex structures with n = 1, 2, 3… rows of free PV stacks between the vortex-chains are indictated by dashed lines labelled with the number of PV rows.

## Discussion

The non-monotonic dependence of γ_eff_ on H_c_ shown in the inset of Fig. 2(i) directly reflects the balance between attractive and repulsive vortex interactions in our system. For very small values of H_c_ dilute linear arrays of PVs are trapped on JV stacks and the attractive JV-PV interaction is maximised by having the most JV/PV crossing points. This drives a contraction of the c-axis JV lattice spacing, c_z_, and a concomitant expansion in the a-b plane, c_y_, leading to the observed increase in γ_eff_. However, as H_c_ is increased, PV stacks on the chains get closer together and a point is reached near the peak in γ_eff_(H_c_), where their mutual repulsion begins to dominate over the JV/PV attraction. This in turn drives a transformation of the JV lattice to the structure with the biggest β $=\,2\sqrt{3}$ (and smaller c_y_ and γ_eff_) and a larger minimum PV-PV spacing and hence lower repulsive interaction. Our detailed measurements of c_y_ as a function of H_c_ at fixed values of H_ab_ are in good qualitative agreement with the theoretical model (see Supplementary Materials), showing a more rapid reduction in chain spacing with H_c_ at lower values of in-plane field and a saturation of c_y_ towards a value that is approximately independent of H_ab_ at large out-of-plane field. In practice, we would not expect the model to match our experiments exactly because it only describes weakly-deformed PV stacks and does not take into account thermal fluctuations. These are expected to renormalise several energy contributions that are described in the Supplementary Materials: the JV-PV crossing energy, the attractive energy of PV stacks, and the interaction energy between neighbouring chains. In general, when thermal displacements are smaller than the separation between PVs in the same layer, thermal fluctuations are only expected to have a small influence on the lattice structure. However, their role will be larger at higher values of H_c_. We do not expect thermal fluctuations to qualitatively modify the theoretical vortex-matter phase diagram, but they may strongly influence the location of transition/crossover lines. The presence of a free sample surface is also not included in our ‘bulk’ model. While we do not expect qualitative changes in the vortex structures near the surface, a reduction of the magnetic interaction between PVs in the stacks in this region should increase deformations caused by JVs and shift the balance towards tilted chains.

At small out-of-plane magnetic fields, when only vortex chains are present, our results are well described by theoretical models for values of the parameter ${\rm{\alpha }}={{\rm{\lambda }}}_{{\rm{ab}}}/{{\rm{\lambda }}}_{{\rm{J}}}$ in the range 0.5 ≤ α ≤ 0.65^11^. For low in-plane fields (H_ab_ < 65 Oe) a small c-axis magnetic field penetrates as structureless chains with an extremely weak magnetic signature, as expected for kinked tilted vortices. Upon increasing H_c_, our results are consistent with the predicted first-order transition to a phase containing chains of pancake-vortex stacks with an approximately ten times higher peak magnetic induction. At still higher c-axis fields, the nucleation of much broader PV stack structures can be associated with the modulated tilted vortices predicted in this regime. Finally, for H_c_ ≥ 3 Oe only bright structureless stripes are observed which we identify with the expected straight tilted vortex phase.

At high out-of-plane fields we also see abrupt step-like increases in the chain spacing which are associated with the formation of particularly stable, ordered composite vortex structures with exactly n = 1, 2, 3… rows of interstitial pancake vortex stacks between the chains. The n = 1 line lies at the boundary between the vortex-chain state at low H_c_ and the composite vortex lattices state at high H_c_ (the grey line running approximately diagonally across Fig. 6). Konczykowski *et al*.^20^ have identifed a line in BSCCO-2212 at very similar magnetic fields on the basis of steps in ac ‘transmissivity’ measured with a micro-Hall sensor. On the assumption that these reflect a transition between a composite state composed of chains of tilted pancake vortices with a single row of free PVs in between to a simple tilted lattice with no free PVs, these authors successfully fit their data to the following theoretical result for the in-plane field at the transition line, ${{\rm{B}}}_{{\rm{ab}}}^{{\rm{t}}}$.

1 $${{\rm{B}}}_{{\rm{ab}}}^{{\rm{t}}}={\rm{C}}\frac{\gamma }{{\lambda }_{{\rm{ab}}}}{[{{\rm{B}}}_{{\rm{c}}}{{\rm{\Phi }}}_{{\rm{0}}}/{\rm{l}}{\rm{n}}(\frac{1.55\sqrt{{{\rm{B}}}_{{\rm{c}}}{{\rm{\Phi }}}_{{\rm{0}}}}}{{{\rm{sB}}}_{{\rm{ab}}}^{{\rm{t}}}})]}^{1/2},$$

where C is a numerical coefficient. For C ≈ 0.043, λ_ab_ = 660 nm and γ = 840, equation (1) is a good fit to the experimental grey line in Fig. 6, confirming that the observed steps in vortex-chain spacing and transmissivity are both signatures of the same vortex transition. The B_c_ − B^t^_ab_ transition line found in ref.^20^ becomes very steep at high in-plane fields and intersects the vortex melting line at a tricritical point. Hence there is a maximum in-plane field at which the transition can be observed, which may explain why we do not see the n = 1 jump at H_ab_ > 450 Oe.

In summary, we have used scanning Hall microscopy to comprehensively map the composite vortex lattices phase diagram in an underdoped BSCCO-2212 single crystal at T = 85 K. Our results are in good semi-quantitative agreement with theoretical models, and show that the effective anisotropy of the Josephson vortex lattice is an unexpected non-monotonic function of out-of-plane field, reflecting a field-driven structural transformation of the JV lattice at high out-of-plane fields. Ultimately, a better understanding of the complex interactions present in the composite vortex matter found in BSCCO-2212 under tilted fields will inform the development of active devices that exploit the intrinsically coupled Josephson and pancake vortex dynamics in this regime, greatly broadening the scope for applications of anisotropic high-T_c_ superconductors. Previously vortex ratchets have been demonstrated based on the response of the vortex system to time-asymmetric in-plane field ‘drives’^21^ and flux lenses realised that exploit the viscous drag of pancake vortices by slowly moving Josephson vortices^22^. Our results clearly identify the optimal operation point for such devices as being near the peak in the effective anisotropy where the response to a change in in-plane magnetic field will be largest and the moving chain states carry the highest linear density of pancake vortex stacks.

## Methods

### Sample Preparation and Measurement Protocol

Our high-quality underdoped BSCCO-2212 single crystal (T_c_ = 89 K) was prepared by the travelling-solvent floating-zone technique^23^. Even the best such crystals contain low levels of quenched disorder capable of pinning vortices in metastable distributions, and to suppress this we employ in-plane field ‘dithering’ which has previously been shown to equilibrate vortex distributions^24^. After application of the desired out-of-plane field at the measurement temperature, the in-plane field is oscillated at a frequency of 200 Hz around the steady state value with an initial amplitude of 6 Oe that decays exponentially to zero with a time constant of 5 s.

### Scanning Hall Microscopy

Here we use scanning Hall microscopy (SHM)^25^ to directly image PVs above the a-b surface of a freshly-cleaved BSCCO-2212 single crystal under independently applied in-plane (H_ab_) and out-of-plane (H_c_) magnetic fields. The SHM used is a modified low-temperature scanning tunnelling microscope (STM) where the tunnelling tip has been replaced by a custom-fabricated semiconductor chip. The Hall probe is patterned in the two-dimensional electron gas of a GaAs/AlGaAs heterostructure, defined by the intersection of two 800 nm wide wires situated ≈5 μm from the Au-coated corner of a deep mesa etch that acts as an integrated STM tip. The Hall probe is mounted at an angle of ≈1° with respect to the sample plane, with the STM tip being the closest point to the sample surface. In operation the sample is first approached towards the sensor until tunnelling is established and then retracted ≈100 nm for rapid ‘flying mode’ scanning with the active Hall probe ≈200 nm above the sample and a spatial resolution of ≈800 nm. In this way quantitative maps of the z-component of magnetic induction can be captured non-invasively. All images shown in this manuscript have been captured at T = 85 K, when the full temperature-dependent scan range of our piezotube is ≈22 μm×22 μm. This temperature is explicitly chosen as it represents the best compromise between improved image contrast and the growing (undesirable) influence of vortex pinning sites as the temperature is lowered. The in-plane field is applied by positioning the scanner head between the poles of a normal electromagnet, and the out-of-plane field is provided by a copper solenoid wound around the tail of the cryostat. Although our imaging technique is only sensitive to PV fields parallel to the crystalline c-axis, the location of stacks of JVs is inferred from the fact that they become “decorated” by high linear densities of PVs due to their mutual attraction.

### Composite vortex lattice theoretical model

To develop a better understanding of the data, we evaluated the chain separation for the ground-state crossing-lattices configurations at different magnetic fields by minimizing the appropriate energy^11^, which is composed of JV lattice, PV-chain, and PV-JV attraction terms (see Supplementary Materials). Figure 4(i) shows the computed chain spacing as a function of B_c_ for several values of B_ab_. Note that we cannot expect an exact match with experiment because the model only describes weakly-deformed PV stacks and does not take into account thermal fluctuations. Nevertheless, this model qualitatively captures the experimental behaviour of Fig. 3 including the increase in chain spacing at very low out-of-plane fields. It also shows a more rapid reduction in chain spacing with B_c_ at lower values of in-plane field, and c_y_ is seen to saturate to a value that is approximately independent of B_ab_ at large out-of-plane field. A careful analysis of these results reveals that the increasing PV density actually drives a structural transformation of the underlying JV lattice. Within London theory the non-interacting JV lattice exhibits two energetically degenerate structures with JV stack spacings ${c}_{y1}=\sqrt{\sqrt{3}\gamma {{\rm{\Phi }}}_{0}/(2{B}_{ab})}$ and ${c}_{y2}=\sqrt{\gamma {{\rm{\Phi }}}_{0}/(2\sqrt{3}{B}_{ab})}\,\,$, respectively. For small out-of-plane fields it is known that the state with the largest JV stack spacing, c_y1_, has the lowest energy because it leads to the largest number of JV-PV crossing points^10^. However, we find that at higher PV densities a transformation to the state with the smaller stack spacing, c_y2_, takes place since this *increases* the on-chain PV-PV spacing and hence reduces the PV-PV repulsion.

### Data availability

All data discussed in this manuscript can be found at 10.15125/BATH-00525.

## Electronic supplementary material

Supplementary Materials
