# Supplementary material for: Tuning the structure of the Josephson vortex lattice in Bi2Sr2CaCu2O8+δ single crystals with pancake vortices
Source: Sci Rep. 2018 Jul 19;8:10914. doi: 10.1038/s41598-018-28681-7 (PMC6053460; doi:10.1038/s41598-018-28681-7)
Supplement: Supplementary file 1 — Supplementary Materials [file 41598_2018_28681_MOESM1_ESM.pdf]

Supplementary Information

Tuning the structure of the Josephson vortex lattice in $Bi_2Sr_2CaCu_2O_{8+\delta}$ single crystals with pancake vortices

P.J. Curran,[1] H.A. Mohammed,[1,2] S.J. Bending,[1*] A.E. Koshelev,[3] Y. Tsuchiya,[4,5] T. Tamegai[4]

[1]Department of Physics, University of Bath, Claverton Down, Bath, BA2 7AY, UK
[2]Department of Physics, University of Kirkuk, Kirkuk, Iraq
[3]Materials Science Division, Argonne National Laboratory, Argonne, Illinois 60439, USA
[4]Department of Applied Physics, University of Tokyo, Hongo, Bunkyo-ku, Tokyo 113-8656, Japan
[5]Department of Electrical Engineering, Nagoya University, Nagoya, Aichi, 4648603, Japan

[*]Correspondence should be addressed to S.J. Bending (email: s.bending@bath.ac.uk)

In this Supplementary Note we provide details for the analytical computations of the equilibrium lattice parameters for the crossing vortex lattices in strongly anisotropic layered superconductors.

I. VORTEX LATTICE PARAMETERS IN TILTED FIELDS IN A STRONGLY ANISOTROPIC LAYERED SUPERCONDUCTOR

A. Set up and energy contributions

We consider crossing vortex lattices appearing in tilted field, which is composed of pancake stacks located on Josephson-vortex rows, see Fig. S1. The lattice parameters are defined in Fig. S1 so that

$$B_z = \frac{\Phi_0}{a c_y},\ B_x = \frac{\Phi_0}{c c_y},\ \frac{a}{c} = \frac{B_x}{B_z}. \tag{1}$$

We only consider the regime $c \ll \lambda$ where $\lambda \equiv \lambda_{ab}$ is the in-plane London penetration depth. Our goal is to evaluate the equilibrium lattice parameters a, c, and c_y for fixed magnetic induction $\boldsymbol{B}$.

The energy per unit volume F per unit volume is approximately given by

$$F = F_{\mathrm{JVL}} + F_{\mathrm{PSL}} + F_{\times}. \tag{2}$$

Here

$$F_{\mathrm{JVL}} = \frac{B_x^2}{8\pi} + \frac{B_x}{\Phi_0}\frac{\varepsilon_0}{\gamma}\left[\frac{1}{2}\ln\frac{c c_y}{2\pi\gamma s^2} + 1.432 + G_L\left(\frac{\gamma c}{4 c_y}\right)\right], \tag{3}$$

is the energy of the Josephson vortex lattice, where γ is the anisotropy factor and $\varepsilon_0 \equiv \Phi_0^2/\left(4\pi\lambda\right)^2$, and the function

$$G_L(r) = \frac{\pi r}{6} - \frac{1}{2}\ln(2\pi r) + \sum_{l=1}^{\infty}\frac{1}{l}\left(\frac{\sinh\left(2\pi r l\right)}{\cosh\left(2\pi r l\right) - (-1)^l} - 1\right)$$

is sensitive to the lattice shape. It has minima at $r = \sqrt{3}/2$ and $1/(2\sqrt{3})$ (corresponding to the two values of the parameter $\beta = 1/r$ introduced in the main text) and the general symmetry property $G_L(1/4r) = G_L(r)$. In the pancake-stack energy F_{PSL} the main contribution is coming from the energy of isolated chains of straight pancake stacks

$$F_{\mathrm{PC}} = \frac{B_z}{\Phi_0}\varepsilon_0\left[\frac{\varepsilon_{PS}^{s}}{\varepsilon_0} + \frac{\pi\lambda}{a} - \ln\frac{4\pi\lambda}{a} + \gamma_E - U\left(\frac{a}{2\pi\lambda}\right)\right], \tag{4}$$

$$U\left(x\right) = \sum_{m=1}^{\infty}\left(\frac{1}{m} - \frac{1}{\sqrt{m^2+x^2}}\right) \approx \begin{cases}\zeta(3)x^2/2, & x \lesssim 0.5\\ \frac{1}{2x} - \ln\frac{2}{x} + \gamma_E - 2\exp(-2\pi x)/\sqrt{x}, & x \gtrsim 1\end{cases},$$

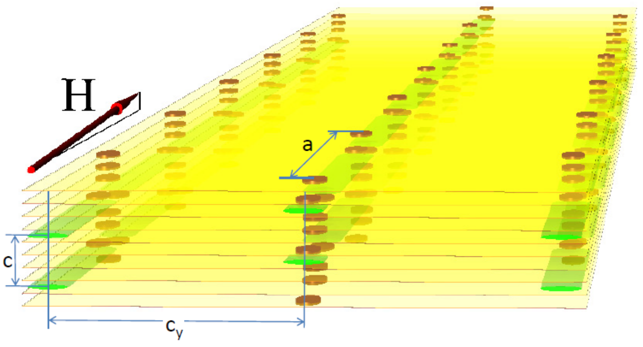

FIG. S1. Lattice parameters of crossing lattices.

where ε_{PS}^{s} is the energy per unit length of an isolated pancake stack and $\gamma_E \approx 0.5772$ is the Euler constant. We also have to take into account the small attractive energy due to pancake-stack deformations

$$F_a \approx -\frac{B_z}{\Phi_0}\varepsilon_0 \frac{16\pi^2}{3} \ln^{-2}\left(\frac{C_u}{\alpha}\right) \frac{s\alpha^2\lambda^2}{ca^2} \tag{5}$$

with $\alpha = \lambda/\gamma s$ and the interaction energy between straight pancake stacks in neighboring chains (we consider the regime $c_y > \lambda$ and neglect more remote interactions)

$$F_i = \frac{B_z}{\Phi_0}\varepsilon_0 \frac{2\pi}{a} \sum_{j=-\infty}^{\infty} \frac{\exp\left[i\pi j - \sqrt{\lambda^{-2} + (2\pi j/a)^2} c_y\right]}{\sqrt{\lambda^{-2} + (2\pi j/a)^2}}. \tag{6}$$

Therefore the total pancake stack contribution is given by

$$F_{\mathrm{PSL}} = F_{\mathrm{PC}} + F_a + F_i \tag{7}$$

Note that the attraction term F_a is only important at very small B_z, while the inter-chain interaction term F_i only becomes important at relatively large B_z. Finally,

$$F_\times \approx -\frac{B_z}{\Phi_0}\varepsilon_0 \frac{8\alpha^2 s}{\ln(3.5/\alpha)c} \tag{8}$$

is the JV-PS crossing energy.

B. Jump in JV spacing c_y caused by penetration of PV stacks

As the PV stacks attract at large separations, their concentration jumps to a finite value corresponding to the finite magnetic induction $B_{z0}(B_x)$. Due to the crossing energy, penetration of the pancake stacks should lead to decrease of the equilibrium vertical separation between the Josephson vortices in comparison with the $B_z = 0$ value. Correspondingly, the horizontal separation c_y should increase. For accurate treatment of this problem we have to consider the energy for fixed H_z and B_x and find equilibrium B_z by minimization of the energy

$$G(H_z, B_x) = \min_{B_z, r_x}\left[F(B_z, r_x, B_x) - \frac{B_z H_z}{4\pi}\right],$$

where $r_x = \frac{c_y}{\gamma c}$ is the parameter related to the aspect ratio of the Josephson vortex lattice. Subtracting irrelevant terms, we introduce the energy function as

$$\tilde{G} = \frac{\gamma\Phi_0}{B_x}\left[F - \frac{B_x^2}{8\pi} - \frac{B_x}{\Phi_0}\frac{\varepsilon_0}{\gamma}\left(\frac{1}{2}\ln\frac{cc_y}{2\pi\gamma s^2} + 1.432\right) - \frac{B_z H_z}{4\pi}\right]/\varepsilon_0. \tag{9}$$

For small B_z and $a \gg \lambda$ we can neglect interaction between pancake stacks in different chains and use exponential asymptotics for the repulsive interaction of straight stacks. In this case $\tilde{G}$ is explicitly given by

$$\tilde{G} \approx G_L(r_x) + \frac{B_z}{B_x}\left[\frac{\varepsilon_{PS}^{s}}{\varepsilon_0} - \frac{\Phi_0 H_z}{4\pi\varepsilon_0} + 2\sqrt{\frac{\pi\lambda}{2a}}\exp(-a/\lambda) - \frac{16\pi^2}{3}\ln^{-2}\left(\frac{C_u}{\alpha}\right)\frac{s\alpha^2\lambda^2}{ca^2} - \frac{8\alpha^2 s}{\ln(3.5/\alpha)c}\right]. \tag{10}$$

Instead of minimization with respect to B_z, it is more convenient to minimize with respect to the reduced lattice parameter $\tilde{a} = a/\lambda$. Introducing notations $\tilde{n}_x = s^2\gamma B_x/\Phi_0$, "chemical potential" $\mu_H = \frac{\Phi_0 H_z}{4\pi\varepsilon_0} - \frac{\varepsilon_{PS}^{s}}{\varepsilon_0}$ and using the relations

$$c = \frac{s}{\sqrt{\tilde{n}_x r_x}}, \frac{B_z}{B_x} = \frac{c}{\gamma\tilde{a}\alpha s} = \frac{1}{\alpha\tilde{a}\sqrt{\tilde{n}_x r_x}},$$

we rewrite the energy function in the reduced form as

$$\tilde{G}(\tilde{a}, r_x) = G_L(r_x) - \frac{8\alpha}{\tilde{a}\ln(3.5/\alpha)} - \frac{16\pi^2}{3}\ln^{-2}\left(\frac{C_u}{\alpha}\right)\frac{\alpha}{\tilde{a}^3} + \frac{1}{\alpha\tilde{a}\sqrt{\tilde{n}_x r_x}}\left[-\mu_H + 2\sqrt{\frac{\pi}{2\tilde{a}}}\exp(-\tilde{a})\right]. \tag{11}$$

We have to minimize this function with respect to $\tilde{a}$ and r_x for different μ_H. In the case when the optimal spacing is at $\tilde{a} = \tilde{a}_m \gg 1$, the minimum conditions are

$$\frac{\partial\tilde{G}}{\partial\tilde{a}} \approx \frac{1}{\alpha\tilde{a}_m^2\sqrt{\tilde{n}_x r_x}}\left[\mu_H + \frac{8\alpha^2\sqrt{\tilde{n}_x r_x}}{\ln(3.5/\alpha)} + \frac{16\pi^2\alpha^2\sqrt{\tilde{n}_x r_x}}{\tilde{a}_m^2\ln^2(C_u/\alpha)} - \sqrt{2\pi a_m}\exp(-\tilde{a}_m)\right] = 0, \tag{12}$$

$$\frac{\partial\tilde{G}}{\partial r_x} = \frac{dG_L}{dr_x} - \frac{1}{2\alpha\tilde{a}_m\sqrt{\tilde{n}_x}r_x^{3/2}}\left[-\mu_H + \sqrt{\frac{2\pi}{\tilde{a}_m}}\exp(-\tilde{a}_m)\right] = 0. \tag{13}$$

Also, at the penetration point we have $\tilde{G}(\tilde{a}_m, r_x) = G_L(r_{x0})$ with $r_{x0} = \sqrt{3}/2$ being the aspect ratio for $B_z = 0$ or

$$G_L(r_x) - G_L(r_{x0}) - \frac{8\alpha}{\tilde{a}_m\ln(3.5/\alpha)} - \frac{16\pi^2}{3}\ln^{-2}\left(\frac{C_u}{\alpha}\right)\frac{\alpha}{\tilde{a}_m^3} + \frac{1}{\alpha\tilde{a}_m\sqrt{\tilde{n}_x r_x}}\left[-\mu_H + \sqrt{\frac{2\pi}{\tilde{a}_m}}\exp(-\tilde{a}_m)\right] = 0. \tag{14}$$

Using this equation, we can transform Eq. (13) into a more convenient form,

$$\frac{dG_L}{dr_x} + \frac{1}{2r_x}\left(G_L(r_x) - G_L(r_{x0}) - \frac{8\alpha}{\tilde{a}_m\ln(3.5/\alpha)} - \frac{16\pi^2}{3}\ln^{-2}\left(\frac{C_u}{\alpha}\right)\frac{\alpha}{\tilde{a}_m^3}\right) = 0 \tag{15}$$

Eqs. (12), (14), and (15) give three equations for three unknowns $\tilde{a}_m$, r_x, and μ_H. Equation (15) allows us to obtain the change of the lattice aspect ratio in the limit $\alpha \ll 1$ when $\tilde{a}_m \gg 1$ and $r_x - r_{x0} \ll 1$. In this case we can use expansion $G_L(r_x) \approx G_L(r_{x0}) + (r - r_{x0})^2/6$ and neglect the last term in Eq. (15) giving

$$r - r_{x0} \approx \frac{12\alpha}{\tilde{a}_m r_{x0}\ln(3.5/\alpha)}.$$

Correspondingly, the change of c_y can be estimated as

$$\frac{c_y - c_{y0}}{c_{y0}} \approx \frac{1}{2}\frac{r_x - r_{x0}}{r_{x0}} \approx \frac{8\alpha}{\tilde{a}_m\ln(3.5/\alpha)}. \tag{16}$$

In more realistic case $\alpha \gtrsim 0.4$, we have to minimize the energy (11) numerically. In this case one has to take into account that the analytic formulas for crossing energy and attractive interaction are valid only in the limit when the separation between the pancake stacks a is much larger than the maximum displacement of pancakes in the crossing region with the Josephson vortex, $u_1 \approx \frac{2.2\lambda^2}{\gamma s\ln(C_u\gamma s/\lambda)}$. To account for this limitation, we introduce phenomenological cutoffs in the corresponding terms in Eq. (11) as $\frac{8\alpha}{\tilde{a}\ln(3.5/\alpha)} \to \frac{8\alpha}{(\tilde{a}+2\tilde{u}_1)\ln(3.5/\alpha)}$ and $\frac{16\pi^2}{3}\ln^{-2}\left(\frac{C_u}{\alpha}\right)\frac{\alpha}{\tilde{a}^3} \to \frac{16\pi^2}{3}\ln^{-2}\left(\frac{C_u}{\alpha}\right)\frac{\alpha}{\tilde{a}^3+8\tilde{u}_1^3}$ with $\tilde{u}_1 = 2\alpha/\ln(C_u/\alpha)$. Suppl. Fig. S2 shows B_x dependences of the relative increase c_y/c_{y0} at the penetration and minimum field $B_{z0} = \Phi_0/c_y a_m$. These curves have been numerically computed assuming $s = 1.56$ nm, $\gamma = 850$, and $\lambda = 0.6$ μm giving $\alpha = 0.45$.

C. Dependences of the lattice parameters on B_z

In this section we compute the field dependences of lattice parameters for fixed B_z larger than $B_{z0}(B_x)$. For the fixed field $\boldsymbol{B}$, due to the relation between the parameters (1), there is only one free lattice parameter, for which we again select the spacing between the pancake stacks in the chain a. We have to minimize energy with respect to this parameter.

To facilitate numerical minimization, we introduce the reduced energy as

$$\tilde{F} = \frac{\Phi_0}{B_z\varepsilon_0}\left[F - \frac{B_x^2}{8\pi} - \frac{B_x}{\Phi_0}\frac{\varepsilon_0}{\gamma}\left(\frac{1}{2}\ln\frac{cc_y}{2\pi\gamma s^2} + 1.432\right)\right] - \frac{\varepsilon_{PS}^s}{\varepsilon_0}$$

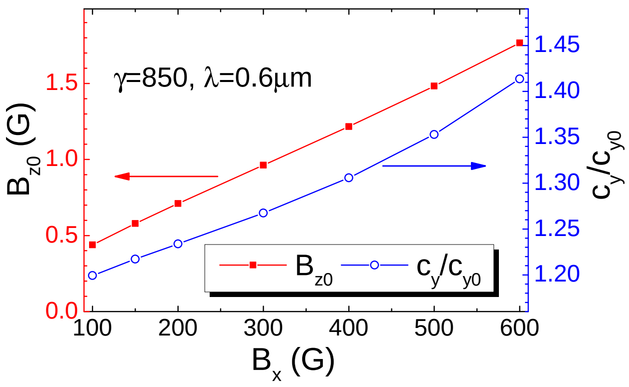

FIG. S2. The B_x dependences of the relative increase c_y/c_{y0} and the minimum field B_{z0} at the pancake-stacks penetration.

and the reduced parameters $\tilde{a} = a/\lambda$, $v_\gamma = a/(\gamma c) = B_x/\gamma B_z$, and $\tilde{B}_z = B_z\lambda^2/\Phi_0$. Then the reduced energy takes the following form

$$\tilde{F} \approx v_\gamma G_L\left(\frac{v_\gamma}{\tilde{a}^2\tilde{B}_z}\right) + \frac{\pi}{\tilde{a}} - \ln\frac{4\pi}{\tilde{a}} + \gamma_E - U\left(\frac{\tilde{a}}{2\pi}\right) - \frac{8\alpha v_\gamma}{\ln(3.5/\alpha)(\tilde{a}+2\tilde{u}_1)} - \frac{16\pi^2}{3}\ln^{-2}\left(\frac{C_u}{\alpha}\right)\frac{\alpha v_\gamma}{\tilde{a}^3+8\tilde{u}_1^3}$$
$$+\frac{2\pi}{\tilde{a}}\exp\left[-1/\left(\tilde{B}_z\tilde{a}\right)\right] + \frac{4\pi}{\tilde{a}}\sum_{j=1}^{\infty}\frac{(-1)^j\exp\left[-\sqrt{1+(2\pi j/\tilde{a})^2}/(B_z\tilde{a})\right]}{\sqrt{1+(2\pi j/\tilde{a})^2}}. \quad (17)$$

We numerically minimized this energy with respect to $\tilde{a}$ and computed the corresponding horizontal spacing as $c_y = \Phi_0/(B_z\lambda\tilde{a})$.

Figure 4(i) of the main text shows computed dependences $c_y(B_z)$ for different B_x. All plots start with minimum B_z at the pancake-stack penetration. At this field c_y always exceeds its value at $B_z = 0$ but rapidly decreases with increasing B_z mostly due to the repulsive interaction between the pancake stacks in the same chain for $a < a_m$. A noticeable feature of these dependences is the regions of quite sharp decrease of c_y within the narrow ranges of B_z. The origin of this phenomenon is the energy structure of the Josephson vortex lattice. It is well known that in the London limit the aligned Josephson vortex lattice is double-degenerate, the function $G_L(r_x)$ has two equal minima at $r_x = \sqrt{3}/2$ and $r_x = 1/(2\sqrt{3})$. At small B_z, due to the crossing energy, the energetically favorable lattice is with larger r_x corresponding to larger c_y. With increasing B_z, due to repulsion between the pancake stacks, eventually the lattice with smaller r_x becomes more favorable. Thus, the rapid decrease of $c_y(B_z)$ marks switching between the two stable configurations of the Josephson vortex lattice.

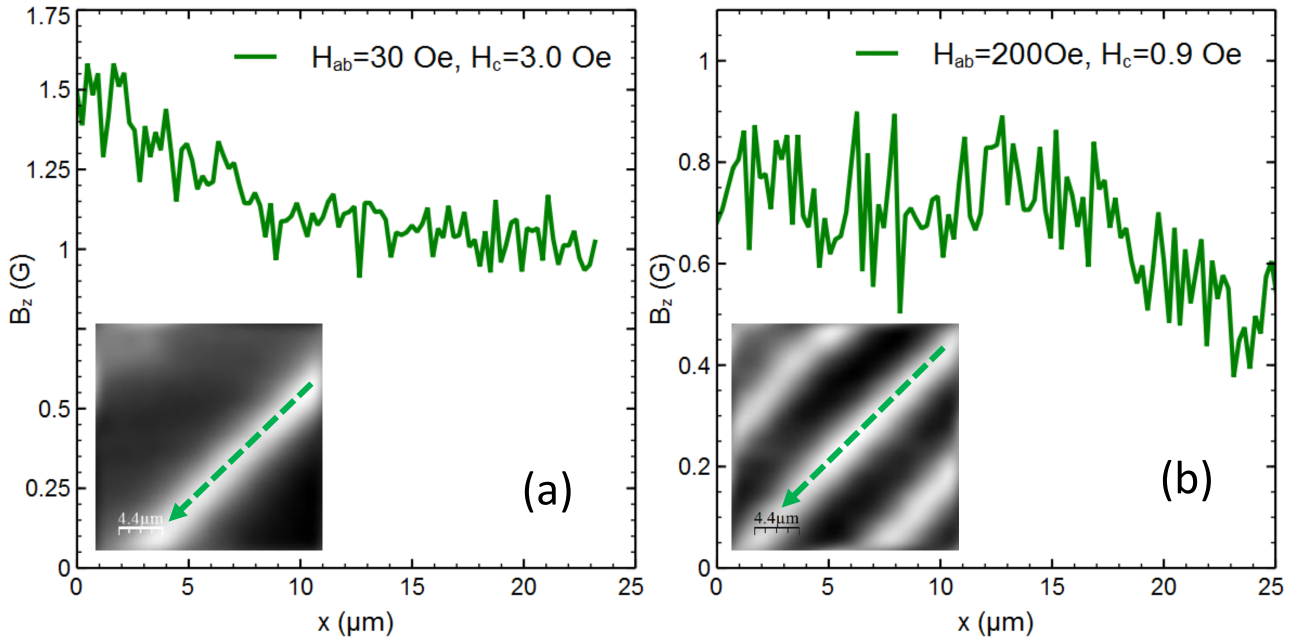

Fig. S3 (a) Linescan along a kinked tilted vortex chain captured at H_{ab}=30 Oe and H_c=3.0 Oe (the direction is indicated by the dashed line in the inserted image). (b) Linescan along the straight tilted vortex chain captured at H_{ab}=200 Oe and H_c=0.9 Oe (the direction is indicated by the dashed line in the inserted image). Within our experimental resolution we do not resolve any modulation due to the periodic array of tilted PV chains.

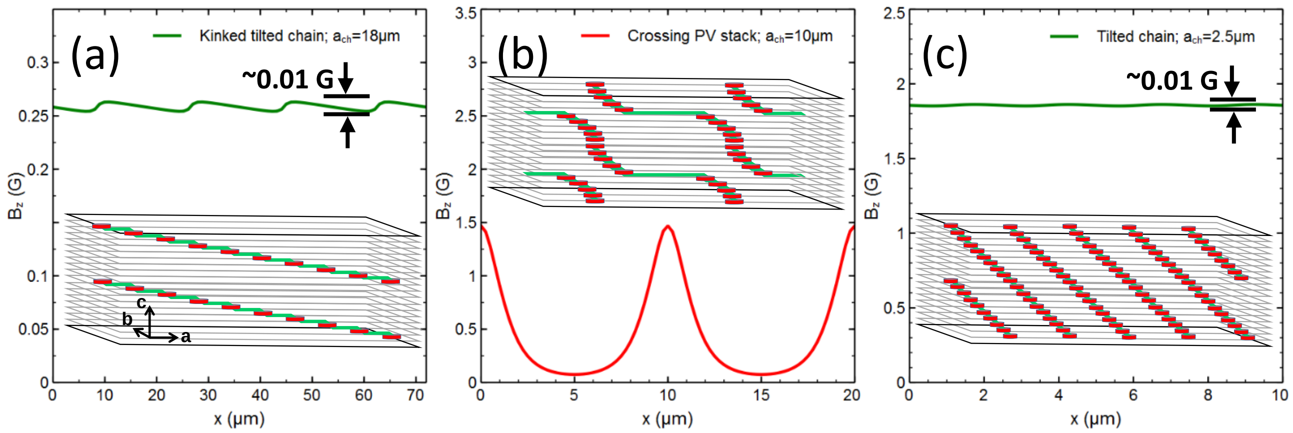

Fig. S4 Simulations of the magnetic induction distribution h=650nm above (a) a kinked tilted chain (c_z=30 nm, a_{ch}=2.5 μm), (b) a well-isolated crossing PV stack (c_z=30 nm, a_{ch}=10 μm) and (c) a straight tilted chain (c_z=30 nm, a_{ch}=2.5 μm). Calculations have been performed using a pancake vortex model derived by John Clem [J.R. Clem, Physica (Amsterdam) **235C**, 2607 (1994) & A.N. Grigorenko *et al.*, Phys. Rev. Lett. **89**, 217003 (2002)]. Calculated linescans along tilted vortices do exhibit an asymmetric modulation due to the periodic arrangement of pancake vortex chains. However, the signal measured at the Hall probe exponentially samples pancake vortices down to a depth of about λ_{ab}=660 nm, and hence averages over many c-axis JV lattice spacings, c_z~30 nm. As a consequence most of the asymmetry due to the tilted vortices is averaged out and the residual modulation of about 0.01 G lies below our measurement resolution (B_{min}~0.1 G with the 100Hz measurement bandwidth used).

The following expression [J.R. Clem, Physica (Amsterdam) **235C**, 2607 (1994) & A.N. Grigorenko *et al.*, Phys. Rev. Lett. **89**, 217003 (2002)] has been used to estimate the magnetic induction along a vortex chain at a height h above the sample surface.

$$B_z(x,h) = \frac{s\Phi_0}{2\pi\lambda_{ab}^2 a_{ch}} \sum_n \sum_{G_i} \int_{-\infty}^{\infty} \frac{\exp(-\sqrt{G_i^2 + q_y^2 + \lambda_{ab}^{-2}}\,ns)\exp(-\sqrt{G_i^2 + q_y^2}\,h)\cos[G_i(x-u_n)]}{\sqrt{G_i^2 + q_y^2 + \lambda_{ab}^{-2}} + \sqrt{G_i^2 + q_y^2}} dq_y \, .$$

Here $G_i=2\pi i/a_{ch}$ are the reciprocal lattice vectors of the chain (a_{ch} is the period of the repeating PV structure along the vortex chain), s=1.5nm is the spacing between CuO_2 bilayers and n is summed over all CuO_2 bilayers down through the thickness of the sample. The set of displacement vectors, u_n, defines the pancake vortex structure with respect to the origin at x=0 within a single period.

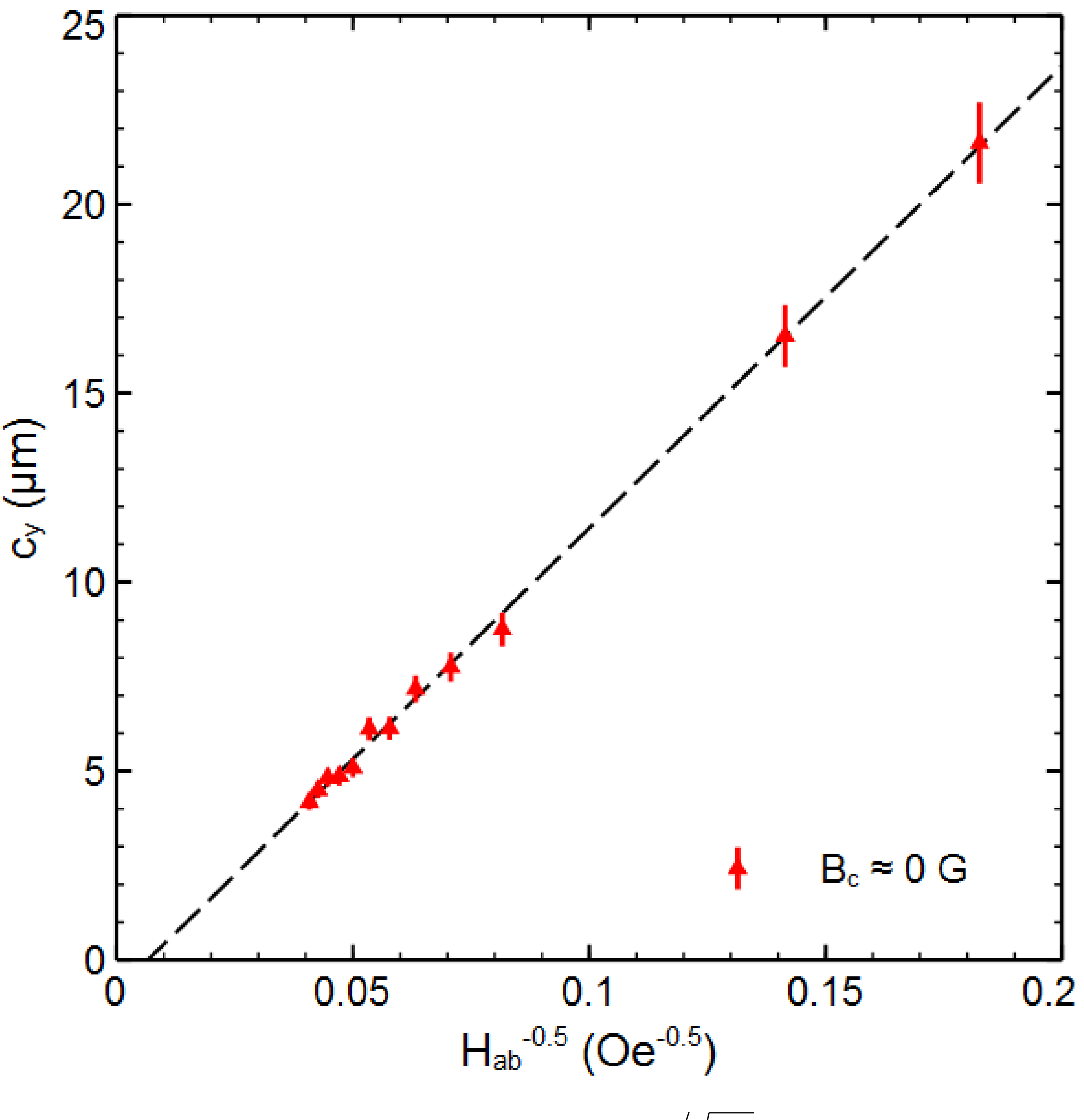

Fig. S5 Plot of the mean vortex-chain spacing, c_y, as a function of $1/\sqrt{H_{ab}}$ at T=85K and the lowest out-of-plane field at which they can be resolved (B_c~0 Oe). The dashed line corresponds to a linear regression fit to anisotropic London theory with γ_{eff}=840±20. Note the excellent linear behaviour exhibited by the data, supporting the assumption that deviation from linearity at higher out-of-plane fields arises due to interactions of Josephson vortices with pancake vortices.
